# Smoking Topography among Korean Smokers: Intensive Smoking Behavior with Larger Puff Volume and Shorter Interpuff Interval

**DOI:** 10.3390/ijerph15051024

**Published:** 2018-05-18

**Authors:** Sungroul Kim, Sol Yu

**Affiliations:** 1 Department of Environment Health Sciences, Soonchunhyang University, Asan 31538, Korea; solsol0914@gmail.com; 2 Division of Environmental Health Research, National Institute of Environmental Research, Incheon 22689, Korea

**Keywords:** topography, cotinine, Fagerstrom score, addiction

## Abstract

The difference of smoker’s topography has been found to be a function many factors, including sex, personality, nicotine yield, cigarette type (i.e., flavored versus non-flavored) and ethnicity. We evaluated the puffing behaviors of Korean smokers and its association with smoking-related biomarker levels. A sample of 300 participants was randomly recruited from metropolitan areas in South Korea. Topography measures during a 24-hour period were obtained using a CReSS pocket device. Korean male smokers smoked two puffs less per cigarette compared to female smokers (15.0 (13.0–19.0) vs. 17.5 (15.0–21.0) as the median (Interquartile range)), but had a significantly larger puff volume (62.7 (52.7–75.5) mL vs. 53.5 (42.0–64.2) mL); *p* = 0.012). The interpuff interval was similar between men and women (8.9 (6.5–11.2) s vs. 8.3 (6.2–11.0) s; *p* = 0.122) but much shorter than other study results. A dose-response association (*p* = 0.0011) was observed between daily total puff volumes and urinary cotinine concentrations, after controlling for sex, age, household income level and nicotine addiction level. An understanding of the difference of topography measures, particularly the larger puff volume and shorter interpuff interval of Korean smokers, may help to overcome a potential underestimation of internal doses of hazardous byproducts of smoking.

## 1. Introduction

The adverse health effects of cigarette smoking are well documented. The rate of death from any cause was 2 to 3 times higher among current smokers as compared to persons who never smoked [1]. In many smoking-related studies, smoking related health effects or the severity of smoking consumption were mainly associated with the duration of smoking, number of cigarettes smoked per day, choice of brand [2,3], cigarette type [4] or topographical factors related to smoking (e.g., puff count per cigarette, puff duration, interpuff interval) [5,6]. 

Recently, one study reported that smokers of “ultra-low/low” nicotine-yield cigarettes exhibited 2.7times more intensive smoking behaviors (*p* = 0.024) to achieve the same salivary cotinine levels as Japanese smokers of “medium/high” nicotine-yield cigarettes [7]. In the United States, a study reported that Korean-Americans tended to exhibit higher average puff flows (*p* = 0.05), greater peak puff flows (*p* = 0.02), and shorter interpuff intervals (*p* < 0.001), compared to Americans of Caucasian descent [8]. Another study found that the higher levels of nicotine metabolites in men relative to women were associated with the puff volumes, as well as the height, weight, and nicotine metabolism of smokers [9]. 

Furthermore, a recent study reported that the delivery of smoking-related carbonyls increased by nearly two-fold when cigarettes were smoked according to the Health Canada Intense (HCI) protocol, compared to the International Organization of Standardization (ISO) method [10]. This finding was consistent with the two-fold difference in total puff volumes between the methods (ISO: 280–315 mL vs. HCI: 495–605 mL). 

A smoker’s topography with a tobacco is highly complex and distinct and the effect of the difference of topographic measures may directly affect to the dose of smoking related byproducts inhaled. As we mentioned above, such variations may be a function of sex, nicotine yield, cigarette type (flavored vs. none flavored) ethnicity, etc. [9,11,12,13].

Although smokers’ compensatory smoking behavior for nicotine-titration were reported in several previous studies [14], explanations for the topographic characteristics of Korean smokers and their association to biomarker levels still have not been fully elucidated. Therefore, this study aimed to evaluate the puffing behaviors of Korean smokers and determine whether smoking-related biomarker levels could be explained by differences in topography measures after controlling for the nicotine addiction level.

## 2. Materials and Methods 

### 2.1. Study Population

The study protocol was approved by the Soonchunhyang (SCH) University Institutional Review Board on March 15, 2016 (no. 1040875-201601-BR-003), and informed consent was received from individuals who agreed to participate in the study. A total of 300 participants (250 men, 50 women) from the Bucheon, Cheonan and Seoul metropolitan areas were recruited into this convenience based study through advertisements at outpatient centers of SCH Bucheon hospitals, in local newspapers, and outside of various commercial establishments (e.g., grocery stores and markets). Self-reported users of nicotine replacement therapy and users of smokeless or chewing tobacco were excluded from the study. Self-reporting pregnant women and individuals with doctor-diagnosed asthma, chronic obstructive pulmonary disease (COPD), or asthma–COPD overlap syndrome were also excluded.

### 2.2. Data Collection

An interviewer-administered questionnaire was used to collect information about demographic characteristics (e.g., age, sex, education level, and race/ethnicity), smoking behaviors (self-reported smoking status, daily smoking amount, nicotine dependence based on the Fagerstrom score, cigarette brand name, number of packages smoked daily), and socioeconomic status. A clinical Research Support System (CReSS) pocket device (BORGWALDT, Richmond, VA, USA) was used to collect data containing the distributions of puff volumes, puff durations, and interpuff intervals from cigarette smoking during a 24-hour period, which was used in many previous studies as a valid tool [6,7,8]. Urine samples were collected from participants who completed topography analyses and were subjected to measurements of cotinine, OH-cotinine, and 4-(methylnitrosamino)-1-(3-pyridyl)-1-butanol (NNAL) concentrations using liquid chromatography-tandem mass spectrometry [15]. Each recipient received U.S. $100 at his/her second visit upon returning the device and providing a urine specimen.

### 2.3. Topography

We used the CReSS Pocket device (a portable version of the CReSS Laboratory system) to collect 24-hour data of puffing behaviors; puff volumes (0–150 mL), puff durations (0–28 s), interpuff intervals (0–1200 s), puff flow (0–150 mL/s), puff counts per cigarette (0–40 puffs), and times to peak puff flow. At Visit 1, each participant received instructions according to the user manual of a CReSS Pocket device and was required to continue using CReSS with their usual brand of cigarettes during the 24 hours. For every usage, each participant was instructed to wait until they heard a beeping sound after inserting a cigarette into the device and to record the number of cigarettes smoked with the device during the 24-hour period. During Visit 2, the pocket device was returned and the data were downloaded. 

After downloading the data, we used a data cleaning procedure (Plowshare Technologies) to identify erroneous puff measurements; if the total number of puffs per cigarette was less than five, we considered the data erroneous and excluded the value from our analysis. Our final topography measures included the puff count, total and average puff volumes (mL), average puff duration (s), average puff flow (mL/Sec), average peak puff flow (mL/Sec), and average interpuff interval (mSec) per cigarette, as well as the number of cigarettes smoked during the recording period (~24 h). The daily total puff volume was calculated by multiplying the median puff volume per cigarette, the corresponding median puff count per cigarette, and the total number of cigarettes smoked using the device during the 24-hour period. The CReSS was calibrated prior to each use by comparing the puff volume of CReSS with a test cigarette connected to a syringe providing suction volume. 

### 2.4. Statistical Analysis

Descriptive statistics (frequencies, percentages, means, medians, and interquartile ranges) were used to characterize the study population and smoking behaviors. Differences in demographics and smoking-related characteristics by sex were determined using Fisher’s exact test and the Mann-Whitney *U* test (Wilcoxon rank-sum test). Statistical significance was defined as a *p* value < 0.05.

A log-transformed linear regression analysis of outcome variables was conducted to account for the right-skewed distribution. The regression coefficients were back-transformed to estimate the geometric mean ratios when comparing different levels of the independent variable. Additionally, we conducted a sensitivity analysis among a subset of the population with low puff counts. As we observed no substantial differences in the findings, the full population is reported herein. Variables that were significant (or borderline significant) in the univariate models were included in the multivariate regression model. All analyses were conducted in SAS version 9.1 (SAS Institute, Cary, NC, USA).

## 3. Results

### 3.1. Demographic Characteristics of the Study Subjects

The demographic characteristics of a total of 250 male smokers and 50 female smokers were summarized by sex in Table 1. The age distributions were similar between male and female subjects, with a majority of subjects younger than 30 years of age. In our sample, 22.0% of women and 50.0% of men had a higher-than-college education level and the difference was significant (*p* = 0.001). Among women, 80% of the subjects had a monthly household income level of $5000 or higher, in contrast to 58.4% of men (*p* = 0.004).

As shown in Table 1, the number of cigarettes smoked per day differed significantly between female and male subjects (*p* = 0.0132). According to the Fagerstrom index, 26.4% and 32.0% of male and female smokers, respectively, were considered to be highly nicotine-dependent (score of ≥5). The difference of the proportion of study participants was not significant (*p* = 0.7676). 

### 3.2. Comparison of Puffing Behaviors between Male and Female Smokers 

**Figure 1:**
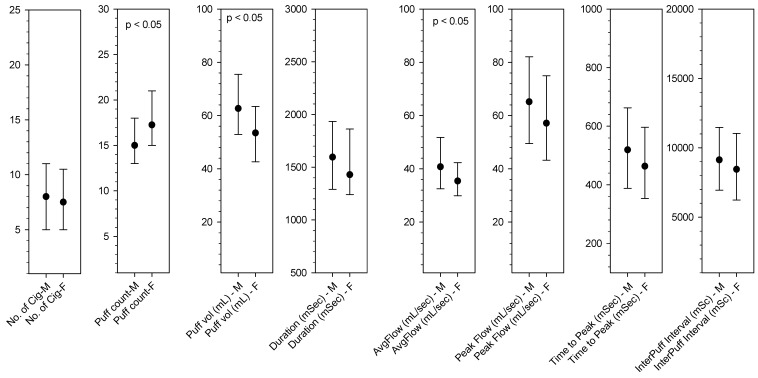
Comparison of puff measures (medina (interquartile range)) between male and female smokers (puff count per cigarette, puff volume, puff duration, average flow rate, peak flow rate, interpuff interval.

In this study, a median of seven cigarettes were smoked using the CReSS device, and this number did not differ significantly by sex (*p* = 0.8143) (Figure 1). Female smokers in our study (17.5 (15.0–21.0)) were likely smoke two puffs more than men (15.5 (13.0–19.0)) (*p* = 0.012) but the puff volumes of women were significantly (*p* = 0.002) smaller than those of men (53.5 (42.0–64.2) mL vs. 62.7 (52.7–75.5) mL). In addition, women had an approximately 10% slower puffing speed than that of men [36.4 (30.0–43.7) mL/s vs. 41.2 (33.1–52.2) mL/s; *p* = 0.0304]. In addition, women had a shorter puff duration (*p* = 0.058) than men, but this was not statistically significant (1.4 (1.2–1.8) s vs. 1.6 (1.2–1.9) s). However, the interpuff interval did not differ significantly between men and women (8.9 (6.5–11.2) s vs. 8.3 (6.2–11.0) s; *p* = 0.122). (Supplementary Table S1).

### 3.3. Comparison of Puffing Behaviors According to Fagerstrom Nicotine Addiction Levels

We also compared the puffing behaviors of subjects stratified by Fagerstrom nicotine addiction levels (low and low-mid (1~4) vs. moderate and high (5 or higher)) (Table 2). Between the two groups, we observed no differences in most puffing behaviors (e.g., puff count per cigarette, puff volume, puff duration, average flow rate, interpuff interval). 

However, according to our further study, those who smoked non-flavored cigarettes were more likely to have higher Fagerstrom scores (i.e., higher nicotine dependence), compared to those who smoked flavored cigarettes (31.2% vs. 13.6%, *p* = 0.0155) (Supplementary Table S2). In addition, the puff count per cigarette was lower (two puffs) among smokers of high-nicotine-dose cigarettes relative to smokers of low-nicotine-dose cigarettes (15.0 (13.0–18.0) times vs. 17.0 (15.0–21.0) times; *p* = 0.0011). In addition, high-nicotine-dose cigarette smokers had a 6.1-mL smaller puff volume relative to low-dose cigarette smokers (59.5 (49.3–71.4) mL vs. 65.6 (54.6–77.4) mL: *p* = 0.0758) (Supplementary Figure S1).

### 3.4. Associations of Daily Total Puff Volume Levels and Urinary Cotinine Concentrations

Supplementary Table S3 divides smokers into four quarters by the daily total puff volume with regard to their quartile value. Here, we found that an increased daily total puff volume was significantly associated with an increased cotinine concentration in a dose-dependent manner (*p* = 0.0011). The median urinary cotinine concentrations among subjects with total puff volumes <4542.0 mL, 4542.1~7071.0 mL, 7071.1~11,556.0 mL, and ≥11,556.1 mL were 905.4 (432.5–1413.2) ng/mL, 933.1 (594.4–1486.2) ng/mL, 1089.9 (787.0–1650.9) ng/mL, and 1271.0 (889.5–1808.9) ng/mL, respectively.

A dose response association of daily total puff volume levels and urinary cotinine concentrations was further evaluated by adjusting for the Fagerstrom index, daily total puff volume (mL), nicotine contents (mg) of a usual cigarette, urinary OH-cotinine concentration, and nicotine (µg/mL) concentration as the major independent variables (Table 3). These associations did not change even after further controlling for sex, age, household income level and the time interval between bio-sample delivery and last cigarette smoked [i.e., recent smoking (min)] (Table 3). Our final multivariate regression analysis showed that the concentrations of urinary cotinine were significantly higher (*p* < 0.05) in groups with high total puff volumes, compared to the reference. Furthermore, we observed a dose-response relationship (*p* < 0.05) between urinary cotinine levels and the Fagerstrom nicotine dependence index. Male smokers were found to have 34% higher cotinine levels relative to female smokers (*p* < 0.05). Although it was not statistically significant, the higher the nicotine content in the cigarettes, the more likely it was to have a positive relationship. The explanatory power of the independent variables in our model is approximately 40%.

## 4. Discussion

The outcome of our study demonstrated similar puff volumes, puff durations, average puff flow rates, and peak flow rates as those obtained from US-dwelling Korean immigrants and American citizens who used the same type of topograph measuring device. However, it also demonstrated that Korean smokers tended to smoke much intensively; the interpuff interval of 8.9 s among Koreans participating in our study was much shorter than that of Americans (25 s) or Korean immigrants living in the USA (13.5 s) [8].

We also found that Korean smokers tend to have strong compensatory smoking behavior for nicotine self-titration. In a recent study of 101 smokers in Japan [7], high-nicotine-dose cigarette users similarly had a lower puff count and puff volume (12.6 times, 53.8 mL). Furthermore, a cross-sectional study conducted in the USA in 2015 observed smaller puff count numbers and puff volumes among high-nicotine-dose cigarette users relative to low-dose cigarette users, although the interpuff intervals were relatively long [16]. 

The International Standard Organization has set a puff volume of 35 mL and interpuff interval of 60 s as the operating parameters of an intense smoking regime [17]. However, according to our study results, we consider that this international setting may not reflect the smoking behaviors of Korean smokers. Health Canada currently uses a puff volume of 55 mL, interpuff interval of 30 s, and puff duration of 2 s. The results of our study demonstrate that the puff volumes and puff durations of Korean smokers were relatively close to the Health Canada guideline, rather than the ISO. However, Korean smokers’ interpuff interval was even 2 to 3 times shorter at 8.9 (6.5–11.2) s for male vs. 8.3 (6.2–11.0) s for female smokers than those from patients of bipolar disorders (18.4 s) [18] or the that of the WHO Standard operating procedure (30 s) for the method of intense cigarette smoking [19].

Our results also demonstrated that male smokers had a lower puff count but higher puff volume relative to female smokers, which might explain the similar median total daily puff volumes of male and female smokers. In other words, the daily total puff volumes did not differ between the sexes, despite differences in various measures of puff parameters (especially volume and frequency). Therefore, we considered that men and women likely inhaled the same amounts of harmful substances if the cigarette type was the same.

Furthermore, our multivariate regression analysis demonstrated that dose-response relationships of the daily total puff volume with the urinary cotinine level did not change even after controlling for and demographic (age, sex), socioeconomic variables (monthly house income level) and the interval between urine sample delivery. We observed that the urinary cotinine concentrations derived for male and female smokers in this study were similar to those reported by the Korea National Health and Nutrition Evaluation study [20]. Therefore, we considered that our results reflected general amount of smoking and its puffing characteristics.

However, a similar multivariate regression analysis of the influencing factors of NNAL found no association with the daily total puff volume (data not shown). We attribute this difference between the biomarkers to the differences in the half-lives of NNAL (~1 month) and cotinine (~1 day) or heterogeneity in the levels of toxins (e.g., NNK) or nicotine (mg) among Korean cigarette brands [21].

A recent study reported that more intensive puffing regimens associated with the use of low nicotine concentration e-liquids could lead to higher levels of carbonyl generation in the aerosol [22]. Furthermore, it was reported that in electronic cigarettes, nicotine consumption and puffing topography could be changed due to the power setting of electronic cigarettes [23]. As the prevalence of e-cigarette use among adult smokers (older than 19 years) had increased from 2013 to 2015 (0.9% to 2.6%) in South Korea while that of tobacco cigarette use had decreased from (19.3% to 17.5%) according to Korea National Health and Nutrition Examination Survey (KNHANES) data (2013–2015) [24], a new topography study with new E-cigarette or heat-not-burn (HNB) cigarette is highly recommended.

We note that our study had some limitations, and therefore our results should be interpreted cautiously. This study population was not nationally representative, but rather involved a convenience-based random sample of volunteers who were solicited via advertisements and agreed to participate. We had a relatively small sample of daily smokers, which limited the multivariate analysis and the ability to assess interactions. Although the subjects were relatively small and not representative of the population, we assumed smoking rates of approximately 40% and 8% among men and women, respectively, and thus aimed to include a number of male smokers (*n* = 250), approximately 5 times larger than female smokers (*n* = 50). As mentioned above, the levels of urinary cotinine were ultimately similar to those reported in the Korea National Health and Nutrition Survey. Therefore, we do not believe that systemic errors or selection bias occurred in terms of our study population recruitment and outcomes. 

When we conducted our study, a small number of the participants reported that they had used electronic cigarettes and common cigarettes together (*n* = 3 out of 300), but not during the study period, and therefore topographical data of electronic cigarettes could not be presented. Furthermore, in this study, according to Tukey’s fences outline test, those subject (*n* = 14) reported more than 30 puffs per cigarettes were considered anomalous outliers and excluded from the final multivariate regression analysis [25]. However, we observed no differences in the distributions (median with interquartile range) of puffing parameters, including the daily total puff volume, before and after excluding the outliers. Finally, in South Korea, there is no exact definition distinguishing low yield cigarettes from reduced nicotine cigarettes. Therefore, it limits further exploration of the differences in smoking behaviors between the users of each cigarette type.

## 5. Conclusions

Despite these limitations, our study provided quantitative evidence of Korean smokers’ compensatory smoking behavior to reach nicotine titration with much intensive puffing behavior: 3 times faster interpuff intervals than that of international intense smoking regime guidelines obtained from Western populations. The application of the ISO or HCI guideline as a parameter of Korean population’s smoking behavior would underestimate the actual daily total puff volume [up to 200% by the difference in puff volume (35 mL vs. 65 mL: ISO, 55 mL vs. 65 mL: HCI) and 300% by the difference in interpuff interval (30 s vs. 9.2 s: ISO and HCI)]. This may result in underestimation of the inhalation amount of smoking-related chemicals, internal doses, and potential health effects per of smoking. 

## Figures and Tables

**Table 1:** Proportion of study subjects according to age, household income, education level, residential area, marital status, smoking status and Fagerstrom nicotine addiction level.

Characterisic		Overall (*n* = 300)		Male (*n* = 250)		Female (*n* = 50)		*p*-Value *
		*n*	(%)	*n*	(%)	*n*	(%)	
Age (year)	≤24	94	(31.3)	73	(29.2)	21	(42.0)	0.115
	25–29	68	(22.7)	62	(24.8)	6	(12.0)	
	30–39	53	(17.7)	46	(18.4)	7	(14.0)	
	≥40	85	(28.3)	69	(27.6)	16	(32.0)	
Monthly household income	≤$5000	114	(38.0)	104	(41.6)	10	(20.0)	0.004
	>$5000	186	(62.0)	146	(58.4)	40	(80.0)	
Education level	University	136	(45.3)	125	(50.0)	11	(22.0)	0.001
	College	164	(54.7)	125	(50.0)	39	(78.0)	
Residential area	Seoul & Bucheon	256	(85.3)	212	(84.8)	44	(88.0)	0.824
	Chungnam & others	44	(14.7)	38	(15.2)	6	(12.0)	
Marital status	Married	100	(33.3)	85	(34.0)	15	(30.0)	0.583
	Unmarried	200	(66.7)	165	(66.0)	35	(70.0)	
No. of cigarette per day	0~5	35	(11.7)	22	(8.8)	13	(26.0)	0.0132
	6~10	119	(39.7)	100	(40.0)	19	(38.0)	
	11~15	89	(29.7)	78	(31.2)	11	(22.0)	
	16~20	43	(14.3)	38	(15.2)	5	(10.0)	
	>20	14	(4.6)	12	(4.8)	2	(4.0)	
Fagerstrom score	1~2 (low)	91	(30.3)	75	(30.0)	16	(32.0)	0.7676
	3~4 (low-mod)	127	(42.3)	109	(43.6)	18	(36.0)	
	5~7 (moderate)	76	(25.4)	61	(24.4)	15	(30.0)	
	≥8 (high)	6	(2.0)	5	(2.0)	1	(2.0)	

* Chi-square test to compare male’s frequency with female’s one.

**Table 2:** Comparison of puff behavior according to the Fagerstrom nicotine addiction levels.

Puffing characteristic	Fagerstrom Category (Low & Low-mid) (*n* = 218)	Fagerstrom Category (Moderate, High) (*n* = 82)	*p*-Value *
No. Cigarettes with CReSS	7.0 (5.0–20.0)	10.0 (7.0–13.0)	<0.01
Puff count/Cig	15.0 (13.5–18.5)	16.0 (13.0–18.5)	0.3041
Puff vol (mL)/Cig	62.1 (52.1–74.1)	58.2 (47.0–72.3)	0.2362
Duration (mSec)/Cig	1572.4 (1285.4–1928.5)	1573.9 (1254.6–1918.9)	0.8958
Average Flow (mL/Sec)/Cig	40.3 (32.5–50.2)	38.6 (30.8–48.1)	0.2384
Peak Flow (mL/Sec)/Cig	65.1 (49.3–81.5)	61.1 (49.1–79.4)	0.3591
Time to Peak (mSec)/Cig	495.5 (370.5–655.3)	556.1 (405.6–637.8)	0.3591
Inter Puff Interval (mSec)/Cig	8927.6 (7375.6–11,281.1)	9122.6 (6460.0–11,468.1)	0.6943

* *p*-value from Mann-Whitney test.

**Table 3:** Determinants of urinary cotinine concentration; Associations with daily total puff volume.

Variable		Univariate (*n* = 286)			Multivariate 1 (*n* = 286, R^2^ = 0.283)			Multivariate 2 (*n* = 286, R^2^ = 0.398)		
		GM Ratio	95% CL		GM Ratio	95% CL		GM Ratio	95% CL	
Fagerstrom index										
	1~2 (Ref)									
	3~4	1.62 *	1.26	2.08	1.43 *	1.12	1.81	1.27 *	1.02	1.59
	5~7	2.40 *	1.82	3.17	2.01 *	1.52	2.66	1.77 *	1.36	2.28
	8+	2.36 *	1.13	4.92	2.28 *	1.13	4.57	2.06 *	1.08	3.90
Total volume (mL)/day										
	≤4715.0 (Ref)									
	4715.1~7245.0	1.47 *	1.09	1.98	1.53 *	1.17	2.01	1.48 *	1.16	1.90
	7245.1~11791.0	1.81 *	1.35	2.44	1.63 *	1.23	2.14	1.65 *	1.28	2.12
	≥11791.1	2.06 *	1.53	2.77	1.72 *	1.30	2.28	1.64 *	1.26	2.12
Nicotine contents (mg)										
	~0.1 (Ref) ^a^									
	>0.1	1.23	0.96	1.57	1.20	0.95	1.51	1.17	0.95	1.45
OH-cotinine (ug/mL)		1.15 *	1.11	1.18				1.11 *	1.08	1.15
Nicotine (ug/mL)		1.22 *	1.13	1.32	1.20 *	1.11	1.29	1.10 *	1.02	1.18
Sex										
	Female (Ref)									
	Male	1.46 *	1.09	1.96	1.39 *	1.08	1.81	1.34 *	1.06	1.70
Age (years)										
	29 or less (Ref)									
	>29	1.04	0.83	1.29	0.98	0.79	1.21	1.07	0.88	1.30
Household income (10,000 Won)										
	>500 (Ref)									
	500 or less	0.78 *	0.62	0.98	0.79	0.65	0.97	0.80	0.66	0.96
Recent smoking (Min)										
	>30 (Ref)									
	30 or less	1.30 *	1.02	1.66	1.16	0.93	1.45	1.12	0.91	1.37

GM = geometric mean; Ref = reference group; *: *p* < 0.05. A: ~0.1 (Ref): cigarettes with nicotine content of 0.1 mg or less, >0.1: cigarettes with nicotine content lager than 0.1 mg.

## Supplementary Materials

The following are available online at http://www.mdpi.com/1660-4601/15/5/1024/s1, Table S1: Comparison of puff behavior of male and female smokers, Table S2: Comparison of distribution of Fagerstrom score between flavored Composition of and non-flavored cigarette smokers, Table S3: Level of urinary cotinine according to the level of daily total puff volume, Figure S1: Comparison of puff measures (medina (interquartile range)) between smokers of high-nicotine-dose cigarettes and low-nicotine-dose cigarettes (puff frequency per cigarette, puff volume, puff duration, average flow rate, peak flow rate, inter puff interval).
