# Supplementary material for: Smoking Topography among Korean Smokers: Intensive Smoking Behavior with Larger Puff Volume and Shorter Interpuff Interval
Source: Int J Environ Res Public Health. 2018 May 18;15(5):1024. doi: 10.3390/ijerph15051024 (PMC5982063; doi:10.3390/ijerph15051024)
Supplement: Supplementary file 1 [file ijerph-15-01024-s001.pdf]

International Journal of
Environmental Research and Public Health

MDPI

Table S1. Comparison of puff behavior of male and female smokers

	All (n = 300) (Median (IQR))	Male (n = 250) (Median (IQR))	Female (n = 50) (Median (IQR))	p-value (M:F)
No of Cigarette (w/ CReSS)	7.0 (5.0-11.0)	7.0 (5.0-11.0)	7.0 (5.0-10.0)	0.8143
Puff count	16 (13.5-19.0)	15.5 (13.0-19.0)	17.5 (15.0-21.0)	0.0123
Puff vol (mL)	60.6 (49.9-73.9)	62.7 (52.7-75.5)	53.5 (42.0-64.2)	0.0019
Duration (mSec)	1552.8 (1243.0-1908.7)	1575.7 (1243.0-1923.0)	1408.9 (1215.6-1847.0)	0.0635
AvgFlow (mL/sec)	40.4 (32.1-51.1)	41.2 (33.1-52.2)	36.4 (30.0-43.7)	0.0304
Peak Flow (mL/sec)	62.7 (49.4-82.4)	66.1 (50.4-85.1)	59.8 (44.3-75.1)	0.3534
Time to Peak (mSec)	493.9 (371.5-640.1)	503.4 (376.4-645.7)	451.1 (347.2-582.3)	0.122
InterPuff Interval (mSec)	8828.9 (6528.6-11196.1)	8931.7 (6546.7-11281.1)	8343.2 (6206.3-10992.9)	0.122

* Mann-Whitney test to compare male's frequency with female's one

Table S2. Comparison of distribution of Fagerstrom score between flavored Composition of and non-flavored cigarette smokers

		1 (Low)		2 (Low-Mod)		3 (Moderate)		4 (High)		p-value*
		n	(%)	*n*	(%)	*n*	(%)	*n*	(%)	
Flavored	NO Capsule Additive Menthol (n = 234)	63	(26.9)	98	(41.9)	67	(28.6)	6	(2.6)	0.0155
	YES Capsule Additive or Menthol (n = 66)	28	(42.4)	29	(44.0)	9	(13.6)	0	(0)	

* p-value from Chi-square test

Table S3. Level of urinary cotinine according to the level of daily total puff volume

	Vol. per_day_ <=4542.0mL		Vol. per_day) 4542.1~7071.0mL		Vol. per_day 7071.1~11556.0mL		Vol. per_day 11556.1 + mL		p-value*
	p50	(IQR)	p50	(IQR)	p50	(IQR)	p50	(IQR)	
Nicotine content in cigarette (mg)	0.4	(0.2~ 0.5)	0.4	(0.2~ 0.5)	0.4	(0.1~ 0.5)	0.4	(0.1~ 0.5)	0.9177
Total vol (ml)/day	2840.0	(2034.6~ 3862.3)	5964.0	(5134.5~ 6448.0)	8672.5	(7728.5~ 10166.4)	16474.5	(12816.9~ 19534.4)	<0.001
Cotinine (ng/mL)	905.4	(432.5~ 1413.2)	933.1	(594.4~ 1486.2)	1089.9	(787.0~ 1650.9)	1271.0	(889.5~ 1808.9)	0.0011

*p-value from Kruskal-Wallis Test

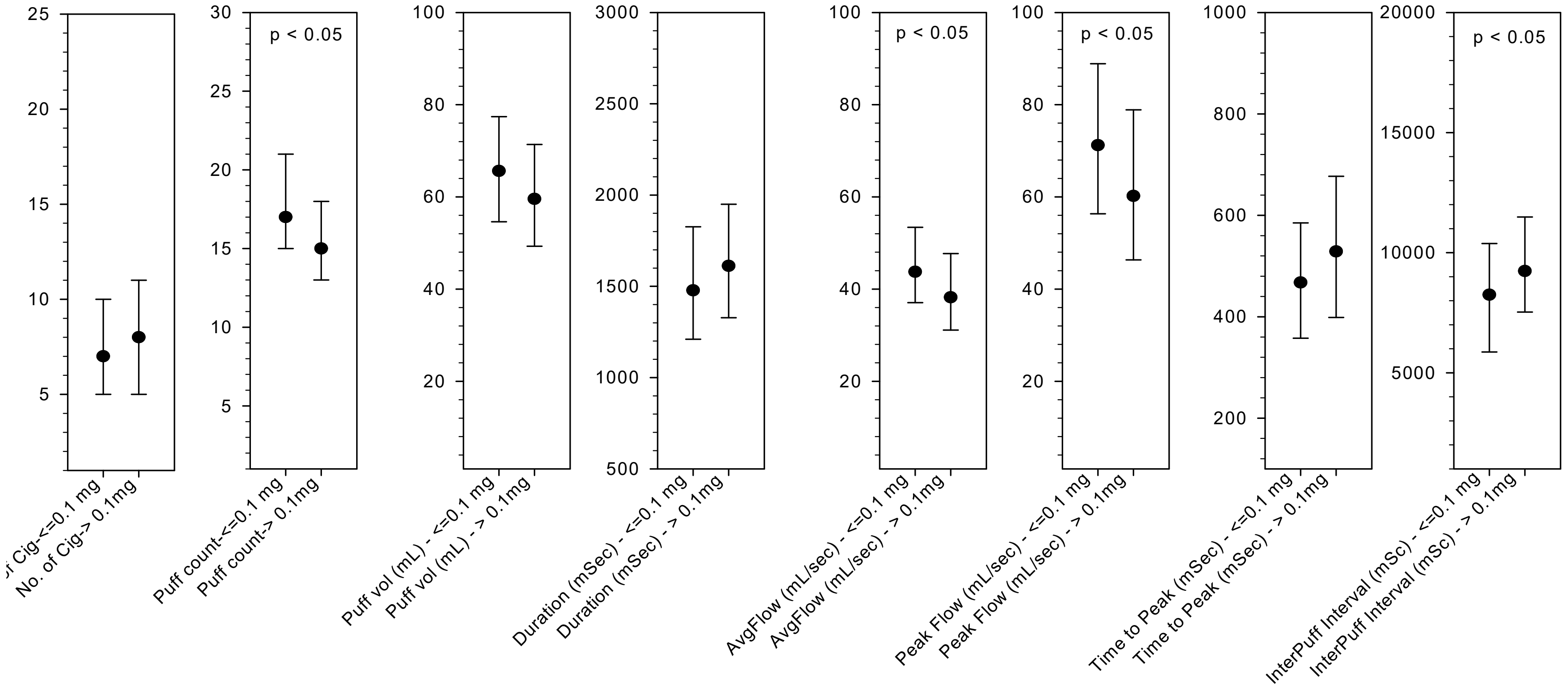

Figure S1. Comparison of puff measures (medina (interquartile range)) between m smokers of high-nicotine-dose cigarettes and low-nicotine-dose cigarettes (puff frequency per cigarette, puff volume, puff duration, average flow rate, peak flow rate, inter puff interval)
